# Chlorogenic acid prevents ovariectomized-induced bone loss by facilitating osteoblast functions and suppressing osteoclast formation

**DOI:** 10.18632/aging.205635

**Published:** 2024-03-09

**Authors:** Chien-Yi Ho, Chih-Hsin Tang, Trung-Loc Ho, Wen-Ling Wang, Chun-Hsu Yao

**Affiliations:** 1 Department of Biomedical Imaging and Radiological Science, China Medical University, Taichung 40202, Taiwan; 2 Division of Family Medicine, China Medical University Hsinchu Hospital, Hsinchu 30272, Taiwan; 3 Physical Examination Center, China Medical University Hsinchu Hospital, Hsinchu 30272, Taiwan; 4 Department of Medical Research, China Medical University Hsinchu Hospital, Hsinchu 30272, Taiwan; 5 Department of Pharmacology, School of Medicine, China Medical University, Taichung 40202, Taiwan; 6 Chinese Medicine Research Center, China Medical University, Taichung 40202, Taiwan; 7 Graduate Institute of Biomedical Sciences, China Medical University, Taichung 40202, Taiwan; 8 School of Post-Baccalaureate Chinese Medicine, China Medical University, Taichung 40202, Taiwan; 9 Department of Chinese Internal Medicine, China Medical University Hospital, Taichung 40447, Taiwan; 10 Department of Chinese Medicine, China Medical University Hospital Taipei Branch, Taipei 11449, Taiwan; 11 School of Chinese Medicine, China Medical University, Taichung 40202, Taiwan; 12 Department of Bioinformatics and Medical Engineering, Asia University, Taichung 41354, Taiwan

**Keywords:** chlorogenic acid, osteoporosis, ovariectomized, osteoclast, osteoblast

## Abstract

Osteoporosis is a usual bone disease in aging populations, principally in postmenopausal women. Anti-resorptive and anabolic drugs have been applied to prevent and cure osteoporosis and are associated to a different of adverse effects. Du-Zhong is usually applied in Traditional Chinese Medicine to strengthen bone, regulate bone metabolism, and treat osteoporosis. Chlorogenic acid is a major polyphenol in Du-Zhong. In the current study, chlorogenic acid was found to enhance osteoblast proliferation and differentiation. Chlorogenic acid also inhibits the RANKL-induced osteoclastogenesis. Notably, ovariectomy significantly decreased bone volume and mechanical properties in the ovariectomized (OVX) rats. Administration of chlorogenic acid antagonized OVX-induced bone loss. Taken together, chlorogenic acid seems to be a hopeful molecule for the development of novel anti-osteoporosis treatment.

## INTRODUCTION

Osteoporosis is a usual bone disorder in aging populations, especially in postmenopausal women [1]. It causes an inhibition in bone mineral density (BMD) and microarchitectural deterioration of bone tissue, enhancing the possibility of bone fragility and fractures. The decrease in estrogen levels leads to increased osteoclast ability and bone resorption, resulting in accelerated bone loss [2]. Anti-resorptive drugs (such as bisphosphonates, denosumab, raloxifene, and calcitonin) and anabolic drugs (like teriparatide and strontium ranelate) have been used to prohibit and treat osteoporosis [3]. Nevertheless, long-term use of these drugs inhibits osteoblast functions and suppresses bone formation by decreasing bone remodeling [4]. Thus, it is important to find effective agents for preventing and treating osteoporosis at a low cost and with minimal undesirable side effects, even after long-term use.

Various traditional herbal medicines are recognized as safe and cost-effective treatments for musculoskeletal disorders, such as *Rhizoma Drynariae*, *Eucommia ulmoides*, *Fructus Psoraleae*, *Salvia miltiorrhiza*, *Herba Epimedii*, *Commiphora myrrha*, and *Radix Dipsaci* [5–10]. Among these, *Eucommia ulmoides* Oliver, commonly called Du-Zhong or Tu-Chung, is a liver- and kidney-tonifying herb [11]. It has been used in East Asia for a long history to treat various diseases, including muscle pain, knee pain, lower back pain, bone fractures, hypertension, hyperglycemia, hyperlipidemia, and joint, liver, kidney, and spleen diseases [12–15]. Regarding to the theory of Traditional Chinese Medicine, the kidney dominates the bone [16]. Thus, Du-Zhong is usually applied in China to strengthen bones, regulate bone metabolism, and treat osteoporosis [17]. The Du-Zhong extract exhibited osteoprotective effects through enhancing osteogenesis and inhibiting osteolysis [17]. It has also been reported that the differentiation of osteoclasts and osteoblasts could be regulated by Du-Zhong bark extract [18]. Application of Du-Zhong cortex extract in adult ovariectomized (OVX) rats inhibited trabecular bone loss, maintained trabecular microarchitecture, and enhanced bone biomechanical quality [18].

Chlorogenic acid is a major polyphenol in many traditional medicinal plants, including Du-Zhong, *Artemisia capillaris*, and *Flos Lonicerae Japonicae* [19]. Moreover, it is widely found in various plants, vegetables, and fruits [19]. Chlorogenic acid possesses several biological and pharmacological functions, such as antioxidant [20], antibacterial, anti-inflammatory [21], anti-carcinogenic, anti-apoptotic [22], anti-obesity [23], hypoglycemic, and hypolipidemic activities. Chlorogenic acid and hyperoside from the hydroethanolic extract of *Artemisia capillaris* inhibited osteoclast formation and bone resorption [24]. Han et al., documented that chlorogenic acid protected osteoblasts against oxidative damage by modulating the PI3K/Akt signaling and increasing Nrf2/HO-1 expression [25]. Moreover, it had an inhibitory effect on dexamethasone-enhanced apoptosis in osteoblasts by promoting the Nrf2/HO-1 anti-oxidative signaling [26]. However, the detailed effects of chlorogenic acid on bone cells are largely unknown. Here, we determined that chlorogenic acid promotes osteoblastic functions and inhibits osteoclast formation *in vitro*. Additionally, treatment with chlorogenic acid antagonized OVX-facilitated bone loss *in vivo*. Taken together, chlorogenic acid appears to be a promising candidate for the management of osteoporosis.

## RESULTS

### Chlorogenic acid enhances osteoblast proliferation and mineralization

**Figure 1:**
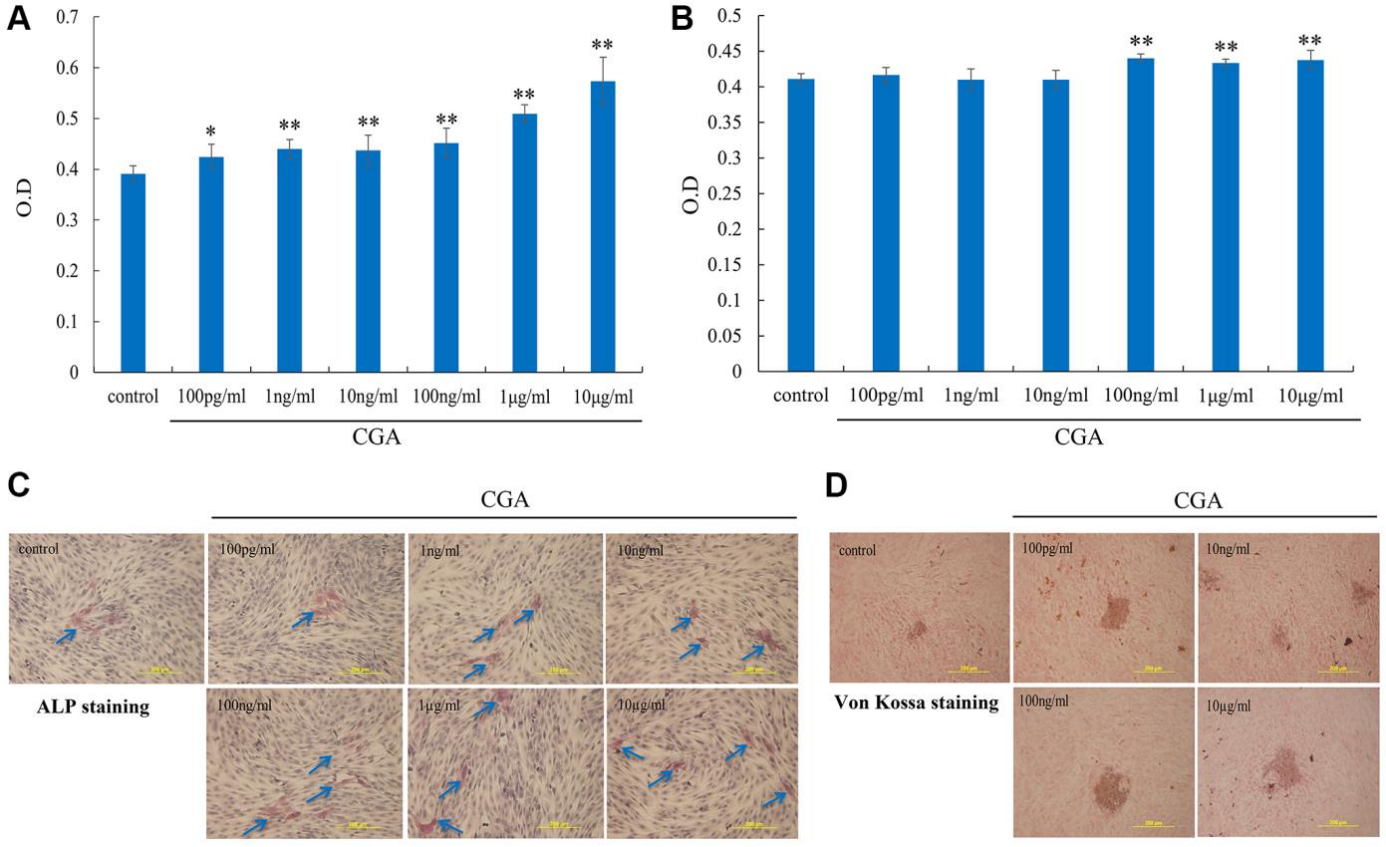
**Chlorogenic acid increases proliferation and differentiation in osteoblasts.** (**A**) MG-63 cells were treated indicated concentration of chlorogenic acid for 2 days, the cell viability was examined by MTT assay. (**B**) MG-63 cells were treated indicated concentration of chlorogenic acid for 2 days, the ALP activity was examined by ALP activity kit. MG-63 cells were incubated with osteoblast differentiation medium (vitamin C (50 μg/mL) and β-glycerophosphate (10 mM)) and chlorogenic acid for 3 and 21 days, the bone mineralization was examined by ALP (**C**) and von Kossa (**D**) staining. ^*^p < 0.05 vs. control group.

The proliferation of osteoblasts enhanced by various concentrations of chlorogenic acid was evaluated by MTT assay after 2 days of culture. Chlorogenic acid significantly promoted the proliferation of osteoblasts at the concentrations from 0.1 ng/mL to 10 μg/mL (Figure 1A). The enzymatic activity of ALP, an early marker of osteogenesis, was determined with an ALP assay kit. Stimulation of MG-63 cells with chlorogenic acid for 2 days facilitated ALP activity in a dose-dependently (Figure 1B). In addition, after culturing MG-63 cells with osteoblast differentiation medium, ALP and von Kossa staining indicated that chlorogenic acid enhanced bone nodule formation (Figure 1C, 1D).

### Chlorogenic acid inhibits osteoclastogenesis

**Figure 2:**
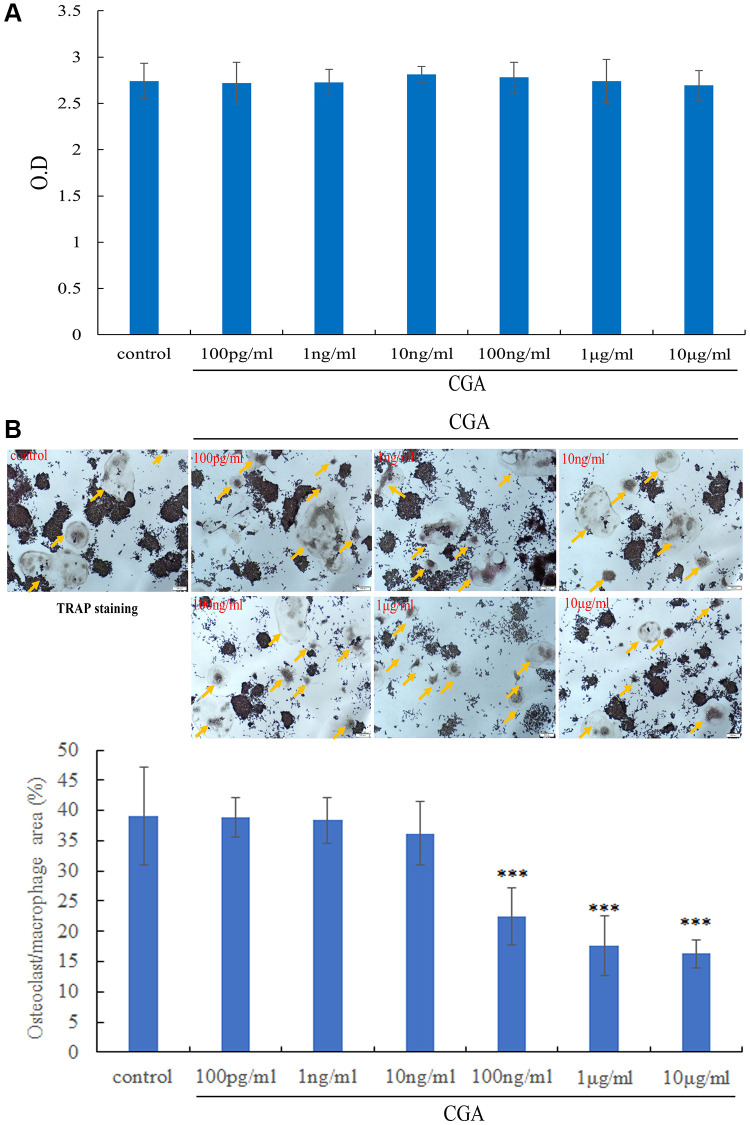
**Chlorogenic acid inhibits osteoclastogenesis.** (**A**) RAW264.7 cells were treated indicated concentration of chlorogenic acid for 2 days, the cell viability was examined by MTT assay. (**B**) TRAP staining and osteoclast number after treating RAW264.7 cells with RANKL and chlorogenic acid for 7 days. ^*^p < 0.05 vs. control group.

Next, we investigate the roles of chlorogenic acid on osteoclast differentiation. Stimulation of RAW264.7 cells with chlorogenic acid at various concentrations had no discernible cytotoxic effects, as determined by the MTT assay (Figure 2A). RAW264.7 cells applied to RANKL for 6 days formed large, mature osteoclasts with multiple nuclei. Osteoclast recognition was featured on phenotypic markers of maturity, for instance TRAP (Figure 2B). Chlorogenic acid significantly reduced osteoclast differentiation, as indicated by staining with the osteoclast marker TRAP (Figure 2B).

### Chlorogenic acid prevents OVX-induced bone loss

**Figure 3:**
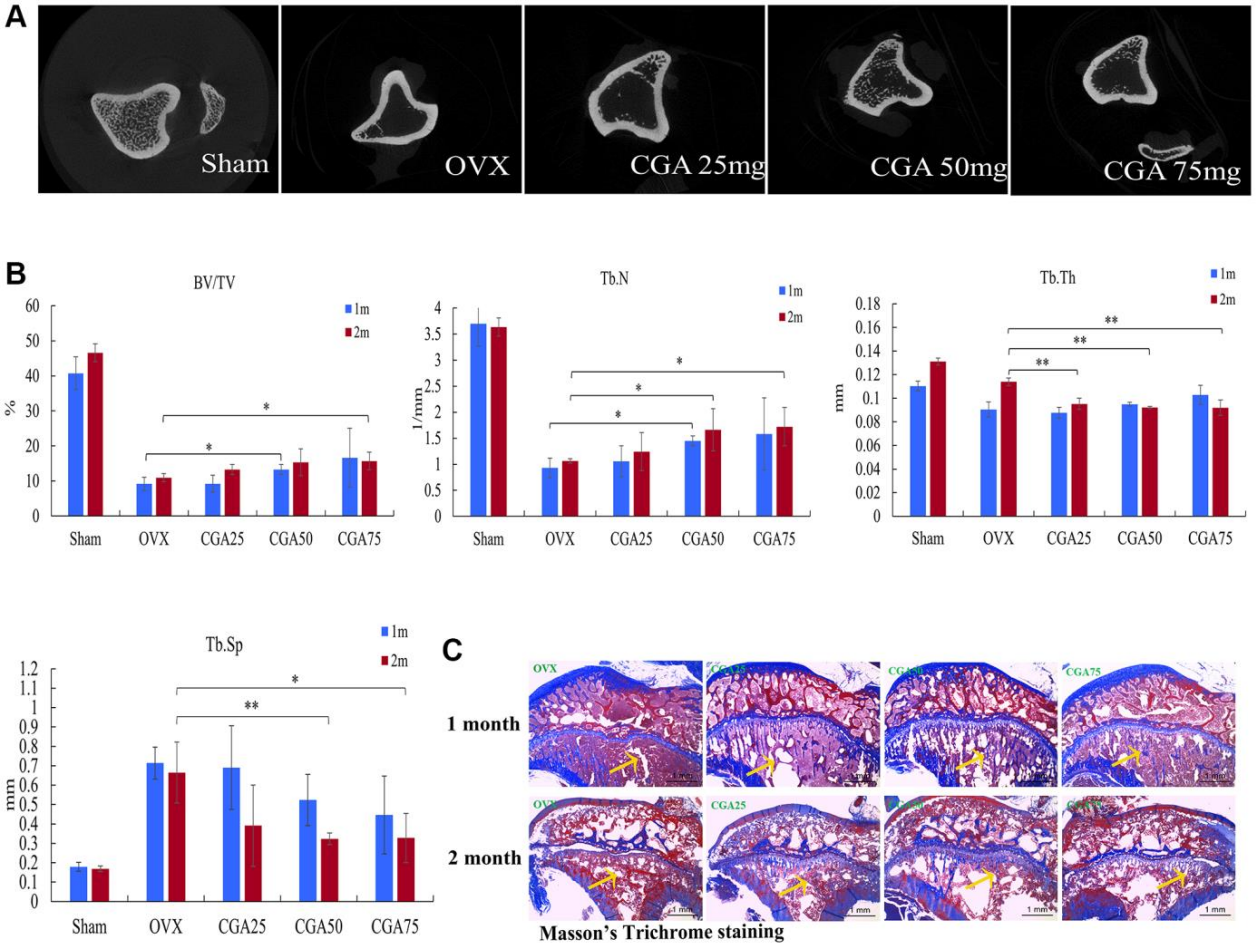
**Chlorogenic acid prevents OVX-induced bone loss.** (**A**) Photomicrographs showing axial views of micro-CT images. (**B**) Graphic illustrations of bone volume fraction (BV/TV), trabecular number (Tb. N.), trabecular thickness (Tb. Th.) and trabecular separation (Tb. Sp.) in the indicated groups. (**C**) Histological sections from tibia stained with Masson’s. ^*^p < 0.05 vs. OVX group.

To examine the function of chlorogenic acid in preventing bone loss *in vivo*, we used the OVX-facilitated osteoporosis model. The micro-CT data demonstrated that OVX inhibited BV/TV, Tb. N., Tb. Th., and enhanced Tb. Sp. However, administration of chlorogenic acid reversed OVX-mediated bone loss (Figure 3A, 3B). Staining using Masson’s confirmed that the enhancement in bone volume was more pronounced in the chlorogenic acid group than in the OVX group (Figure 3C).

**Figure 4:**
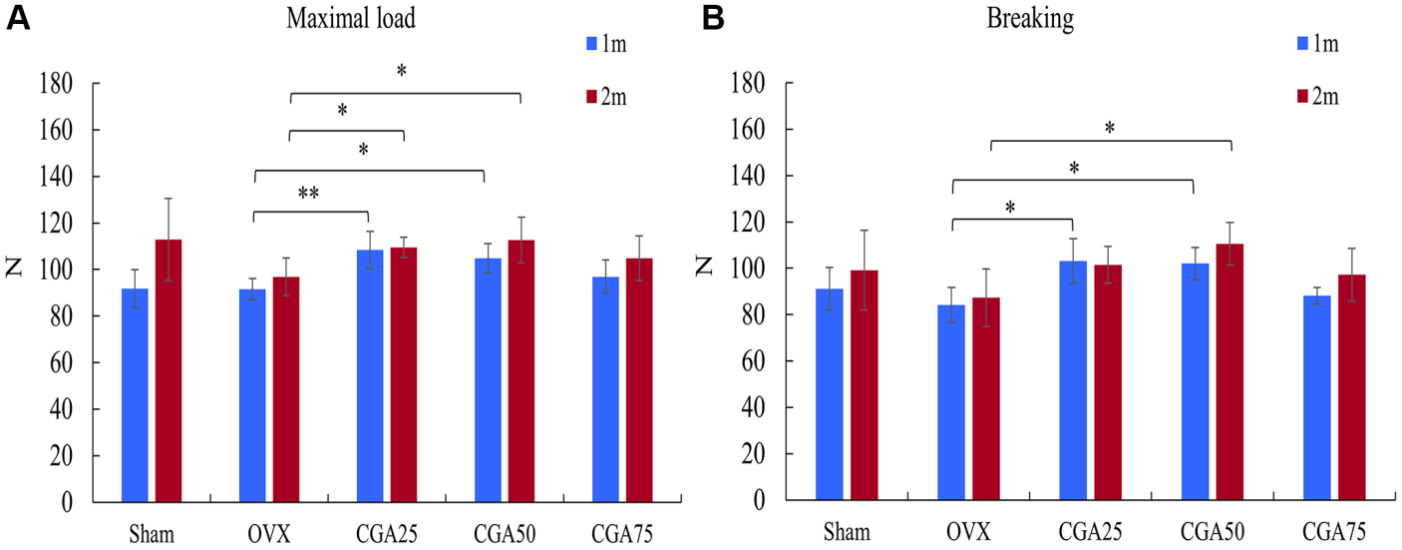
**Chlorogenic acid reversed OVX-reduced biomechanical properties.** The mechanical properties of femurs were evaluated by three-point bending tests. (**A**) Loading force to maximal load. (**B**) Loading force to tissue fracture. ^*^p < 0.05 vs. OVX group.

According the three-point bending test, the OVX rats displayed the lowest maximal and fracture load (Figure 4A, 4B). Chlorogenic acid markedly reversed OVX-reduced maximal and fracture load (Figure 4A, 4B).

## DISCUSSION

Du-Zhong has numerous therapeutic benefits, including pain treatment, bone fractures, hypertension, hyperglycemia, and anti-inflammation [12–15]. Du-Zhong seed extract has been shown to increase the BMD and bone strength of the femur in normal rats [27]. The BMD of the tibiae and femora in OVX rats significantly increased after being fed with Du-Zhong leaf extract [28]. Additionally, Du-Zhong cortex extract has been indicated to reduce disuse- and lead-induced bone loss [29, 30]. Chlorogenic acid is the principal polyphenol compound in Du-Zhong [19]. In the current study, chlorogenic acid was shown to enhance osteoblast proliferation and mineralization. Furthermore, chlorogenic acid also reduces RANKL-facilitated osteoclastogenesis in RAW264.7 cells. Importantly, chlorogenic acid antagonized OVX- facilitated bone loss *in vivo*. Taken together, chlorogenic acid is a strong candidate for the development of anti-osteoporosis remedies.

Oxidative stress may enhance bone resorption and decrease bone formation by promoting osteoclastogenesis and suppressing osteogenesis [31]. Plant-derived polyphenolic compounds exhibit antioxidant ability by scavenging free radicals, diminishing lipid peroxidation, and chelating metal ions. Thus, polyphenolic compounds are considered useful in preventing bone loss. The bark, leaves, and seeds of Du-Zhong contain abundant polyphenolic compounds, including lignans, phenolic acids, and flavonoids [12, 17]. *In vitro* and *in vivo* reports have found that Du-Zhong extract has a strong antioxidant capacity [32]. An ethanol extract of Du-Zhong leaves was demonstrated to suppress the apoptosis of osteoblasts (MC3T3-E1 cells) induced by H_2_O_2_ by inhibiting oxidative stress [19]. Chlorogenic acid has displayed antioxidant ability [33]. In osteoblasts, chlorogenic acid prevented the damaging effects of oxidative stress [25]. Here, we examine the direct effect of chlorogenic acid on osteoblastic cells. We found that chlorogenic acid increases osteoblast proliferation and mineralization. Whether the antioxidant ability of chlorogenic acid also contributes to its promotion of osteoblast functions requires further examination. The limitations should be mentioned with the current study. We examined the osteoblast effects in MG-63 cells (human osteosarcoma cell lines) but not in human osteoblasts. MG-63 cells expressed similar cellular functions such as osteoblastic markers with osteoblasts. However, we don’t have human osteoblast cells to confirm the results in MG-63 cells. Further examination of chlorogenic acid should be performed in human osteoblasts.

Bone health is determined by a dynamic balance of osteoblastic bone formation and osteoclastic bone resorption. Raised bone resorption ability disrupts the aforementioned equilibrium, leading to bone microstructure abnormalities and/or bone impairment in conditions like osteoporosis [34, 35]. RANKL is the major pathogenic mediator that causes bone impairment by increasing bone resorption and subsequent bone tissue destruction [35, 36]. The expression of osteoclast differentiation factor RANKL was decreased by treatment with Du-Zhong extract, while the expressions of osteoblast differentiation markers, Runx2 and Osterix, were increased [18]. Application of Du-Zhong cortex extract in adult OVX rats inhibited trabecular bone loss, maintained trabecular microarchitecture, and enhanced bone biomechanical quality [18]. In this study, we applied murine macrophages (RAW264.7 cells) to differentiate osteoclasts via *in vitro* RANKL activation with the purpose of assessing the roles of chlorogenic acid on osteoclastogenesis. We found that chlorogenic acid reduced RANKL-induced osteoclast formation, implying that chlorogenic acid possesses anti-resorptive abilities. Therefore, chlorogenic acid could be further developed for use in other bone loss disorders, such as osteoarthritis and periodontitis.

Natural products and synthetic compounds based on natural prototypes have garnered considerable interest due to their biological efficacy and low side effects. Here, we report that chlorogenic acid, a major compound from Du-Zhong, mitigates OVX-induced bone impairment by enhancing osteoblast functions and reducing osteoclast formation. Therefore, chlorogenic acid appears to be a promising candidate for the treatment of osteoporosis. We also suggest that Du-Zhong and chlorogenic acid can be explored as novel therapeutic avenues for addressing bone loss disorders.

## MATERIALS AND METHODS

### Cell culture

The human osteoblast-like cell line MG-63 (BCRC No. 60279) and murine monocyte/macrophage RAW 264.7 cells (BCRC No. 60001) were bought from the Food Industry Research and Development Institute (Hsinchu, Taiwan). MG-63 cells were cultured in Dulbecco’s modified Eagle’s medium (DMEM; Gibco, Grand Island, NY, USA) with 10% fetal bovine serum (FBS; Gibco) at 37°C under a humidified 5% CO_2_ atmosphere.

### MTT assay for cell viability

After treatment with chlorogenic acid, the medium was changed with 50 μL of 3-(4,5-dimethylthiazol-2-yl)-2,5-diphenyltetrazolium bromide (MTT) reagent (5 mg/mL, Sigma-Aldrich) and 500 μL of culture medium. The cells were cultured at 37°C for 4 h to form insoluble formazan crystals, which were dissolved in 500 μL of dimethyl sulfoxide. The concentration of formazan crystals formed in the viable cells was analyzed at a wavelength of 570 and 650 nm using a SpectraMax 340PC384 microplate reader (Molecular Devices, Sunnyvale, CA, USA).

### Analysis of ALP activity

After treatment with chlorogenic acid, the cells were treated at 37°C for 30 min to synthesis *p*-nitrophenol from the hydrolysis of *p*-nitrophenyl phosphate. The alkaline phosphatase (ALP) activity was assessed using ALP assay kit (86R-1KT; Sigma-Aldrich).

### Osteoblast differentiation

MG-63 cells (5 × 10^4^) were cultured in osteoblast differentiation medium (vitamin C (50 μg/mL) and β-glycerophosphate (10 mM)) along with chlorogenic acid for 21 days. Cells were fixed with acetone for 30 seconds then stained with ALP or von Kossa staining reagent using established methods [37, 38].

### Osteoclast differentiation

RAW264.7 cells were applied with receptor activator of nuclear factor kappa beta ligand (RANKL) and chlorogenic acid at different concentrations. After 6 days, multinucleated (N ≥ 3 nuclei) tartrate-resistant acid phosphatase (TRAP)-positive cells were identified as mature osteoclasts, following methods established in a previous study [39].

### OVX-induced osteoporosis model

Sham-operated and OVX Sprague–Dawley female rats at the age of 8 weeks were obtained from LASCO (Yi-Lan, Taiwan). All animal procedures were approved by the Ethical Committee for Animal Experiments at China Medical University (Taichung, Taiwan). Four weeks after OVX or sham operation, the rats were divided into four groups: (1) sham operation without treatment (SO, *n* = 4); (2) OVX without treatment (OVX, *n* = 4); (3) OVX treated with chlorogenic acid at graded doses (CA25, *n* = 4, 25 mg/day), (CA50, *n* = 4, 50 mg/day), (CA75, *n* = 4, 75 mg/day) and (CA100, *n* = 4, 100 mg/day). Chlorogenic acid was dissolved in distilled water and administered orally by tube for 8 weeks, after which the rats were sacrificed by intramuscular injection of a mixture of Zoletil^®^ 50 (Virbac, Carros, France) and Rompun^®^ (Bayer Korea Ltd., Seoul, South Korea).

The right tibia was dissected and fixed in a 10% phosphate-buffered formalin solution (Merck, Whitehouse Station, NJ, USA). The trabecular microarchitecture, including the bone volume fraction (BV/TV), trabecular number (Tb. N.), trabecular thickness (Tb. Th.) and trabecular separation (Tb. Sp.) was measured using microCT (SkyScan-1176, Bruker MicroCT, Aartselaar, Belgium).

### Mechanical testing

The mechanical properties of femurs were evaluated by three-point bending tests using a testing device (RT1-TST, Royalty Tec. Ins. Ltd, Kaohsiung, Taiwan). The femur was clamped between two supporting points with a distance of 25 mm. The load was applied to the middle of the specimen at a rate of 2 mm/min until fracture occurred. The maximum load to failure was obtained from the load-deformation curves.

### Histomorphological analysis

The femurs were fixed in 10% buffered formalin, decalcified in EDTA, embedded in paraffin, and sliced into 5 μm thick coronal sections. The sections were stained with Masson’s staining reagent.

### Statistical analysis

Quantitative data were presented as means ± standard deviations. Statistical analysis was carried out by one-way analysis of variance. Multiple comparisons were performed with Fisher’s least significant difference test. Statistical significance was set when *p*-value was lower than 0.05.
